# Intratumoral microorganisms and artificial antitumor bacteria

**DOI:** 10.1016/j.fmre.2023.06.002

**Published:** 2023-06-17

**Authors:** Wenna Li, Xiaotu Ma, Xiao Zhao

**Affiliations:** a CAS Key Laboratory for Biomedical Effects of Nanomaterials and Nanosafety & CAS Center for Excellence in Nanoscience, National Center for Nanoscience and Technology of China, 11 Beiyitiao, Zhongguancun, Beijing 100190, China; b Center of Materials Science and Optoelectronics Engineering, University of Chinese Academy of Sciences, Beijing 100049, China

**Keywords:** Intratumoral microorganisms, Cancer biology, Antitumor bacteria, Tumor therapy, Synthetic biology

## Abstract

Intratumoral microorganisms have been detected in multiple cancer types, which can significantly affect the tumor progression and antitumor treatment efficacy. Considering the important role of intratumoral microorganisms in cancer development, novel therapeutic and diagnostic approaches based on living bacteria have received increasing attention in oncology. In addition to wild types, bacteria can also be programmed through synthetic biology techniques to enhance the spatial and temporal controllability. Therefore, once perfected, intratumoral microorganisms can be combined with immunotherapy or other powerful antitumor agents and possibly unlock the next generation of precision cancer diagnosis and therapy. Moreover, the current challenges and perspectives have also been discussed.

Human body is a superorganism composed of its own cells and numerous symbiotic microorganisms. The gastrointestinal microorganisms have been shown to affect proximal and even distant tumor biology. For instance, intestinal commensal *Bifidobacterium* evoke anti-melanoma immunity via promoting the infiltration and activation of tumor-specific CD8^+^ T cells, thereby facilitating the efficacy of programmed cell death 1 ligand 1 (PD-L1) blockade therapy [1]. Interestingly, it has recently been discovered that many microorganisms also colonize in the tumor tissues, being termed as intratumoral microorganisms. Due to the closer spatial relationship, their impact on tumors may be more pronounced than the gastrointestinal microorganisms, which is still unclear.

## 1 Presence and discovery of intratumoral microorganisms

Bacteria were first detected in human tumors more than 100 years ago, but these bacteria were considered as contaminants during specimen collection. With the advances in sampling and sequencing technologies, more and more intratumoral microorganisms have been confirmed and identified, including bacteria, fungi, and viruses. The unique physicochemical and biological characteristics of tumor microenvironment (TME) are beneficial to the microorganism colonization and growth, such as the vasculature disorder, the highly hypoxic but nutrient-rich components, and the immunosuppression. It has been shown that the potential sources of intratumoral microorganisms can be classified into different categories, through mucosal barrier sources, from adjacent normal tissues, or hematogenous spread. The most intratumoral microorganisms are intracellularly present in both cancer and immune cells with a low biomass level. At present, intratumoral microorganisms have been found in varieties of cancer types, such as lung, breast, colorectum, pancreatic and ovary tumors. However, there are distinct intratumoral microorganism spectrums in different tumor types, and of course their effects on cancer biology (such as tumor growth, metastasis, physical and immune microenvironment) are also greatly different [2]. The existence of intratumoral microorganisms increases the complexity of cancer biology, and the in-depth exploration of intratumoral microorganisms may provide new strategies for cancer therapy.

## 2 Relationship between intratumoral microorganisms and tumors

**Fig. 1:**
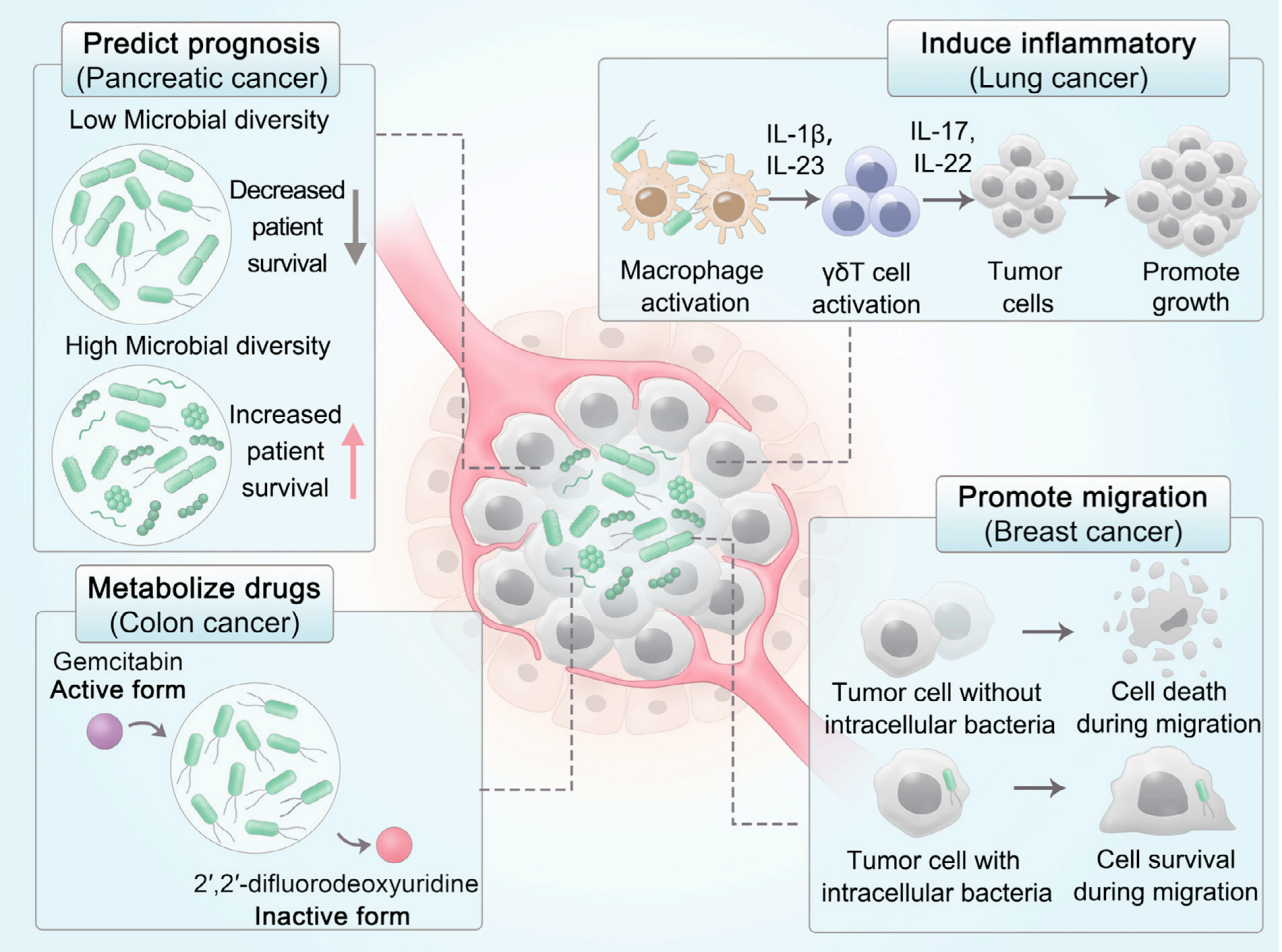
**Relationship between intratumoral microorganisms and multiple types of cancer.** Commensal microbiota can promote lung cancer development by activating lung-resident γδ T cells to secrete inflammatory effector molecules [3]. Intracellularly colonized bacteria can promote metastasis of breast cancer by reorganizing the actin cytoskeleton and enhancing the resistance of tumor cells to fluid shear stress [4]. Intratumoral Gammaproteobacteria can metabolize the chemotherapeutic drug gemcitabine into its inactive form, leading to the drug resistance [5]. Intratumoral microorganisms can affect the tumor progression and the antitumor treatment efficacy through several mechanisms. Pancreatic cancer patients with higher intratumoral microbial diversity have longer median survival, and therefore the microbial diversity can be used to predict patient prognosis [6].

Intratumoral microorganisms can affect the tumor progression and the antitumor treatment efficacy through several mechanisms, such as DNA damage, induction of immunosuppression or inflammatory response, promotion of migration, and metabolization of therapeutic drugs (Fig. 1). For instance, commensal microbiota can promote lung cancer development by activating lung-resident γδ T cells to secrete inflammatory effector molecules [3]. Intracellularly colonized bacteria can promote metastasis of breast cancer by reorganizing the actin cytoskeleton and enhancing the resistance of tumor cells to fluid shear stress [4]. Intratumoral Gammaproteobacteria can metabolize the chemotherapeutic drug gemcitabine into its inactive form, leading to the drug resistance, which is proved by the evidence that the co-treatment of gemcitabine and the antibiotic ciprofloxacin can eliminate the drug resistance [5]. Although the intratumoral microorganisms are mainly reported to promote the tumor progression, they can also inhibit tumor development in certain condition. Pancreatic cancer patients with higher intratumoral microbial diversity have longer median survival, and therefore the microbial diversity can be used to predict patient prognosis [6]. In addition to bacteria, it has been recently found that the intratumoral fungi also possesses close relationship to cancer. The presence of *Malassezia globosa* in breast cancer and the enrichment of *Candida* in gastrointestinal cancer are significantly associated with decreased patient survival [7,8]. In brief, the intratumoral microorganisms exhibit a great influence on tumor progression and therapeutic outcome, thus the comprehensive exploration and artificial manipulation of intratumoral microorganisms may pave the way for novel treatment options for cancer patients.

## 3 Artificial antitumor bacteria

Considering the important role of intratumoral microorganisms in cancer development, novel therapeutic and diagnostic approaches based on living bacteria have received increasing attention in cancer research. Wild-type bacteria can be used for tumor treatment and diagnosis due to their natural tumor-specific targeting and intratumoral penetration capabilities. Certain microorganisms can be employed as efficient vehicles for tumor-targeted drug delivery because they can specifically colonize in tumor tissues. In addition, different types of tumors have their distinct and specific intratumoral microorganism profiles, which are closely associated with patient survival. Therefore, the plasma-derived cell-free microbial nucleic acids have been used as effective tools for early tumor prediction in clinic [9].

**Fig. 2:**
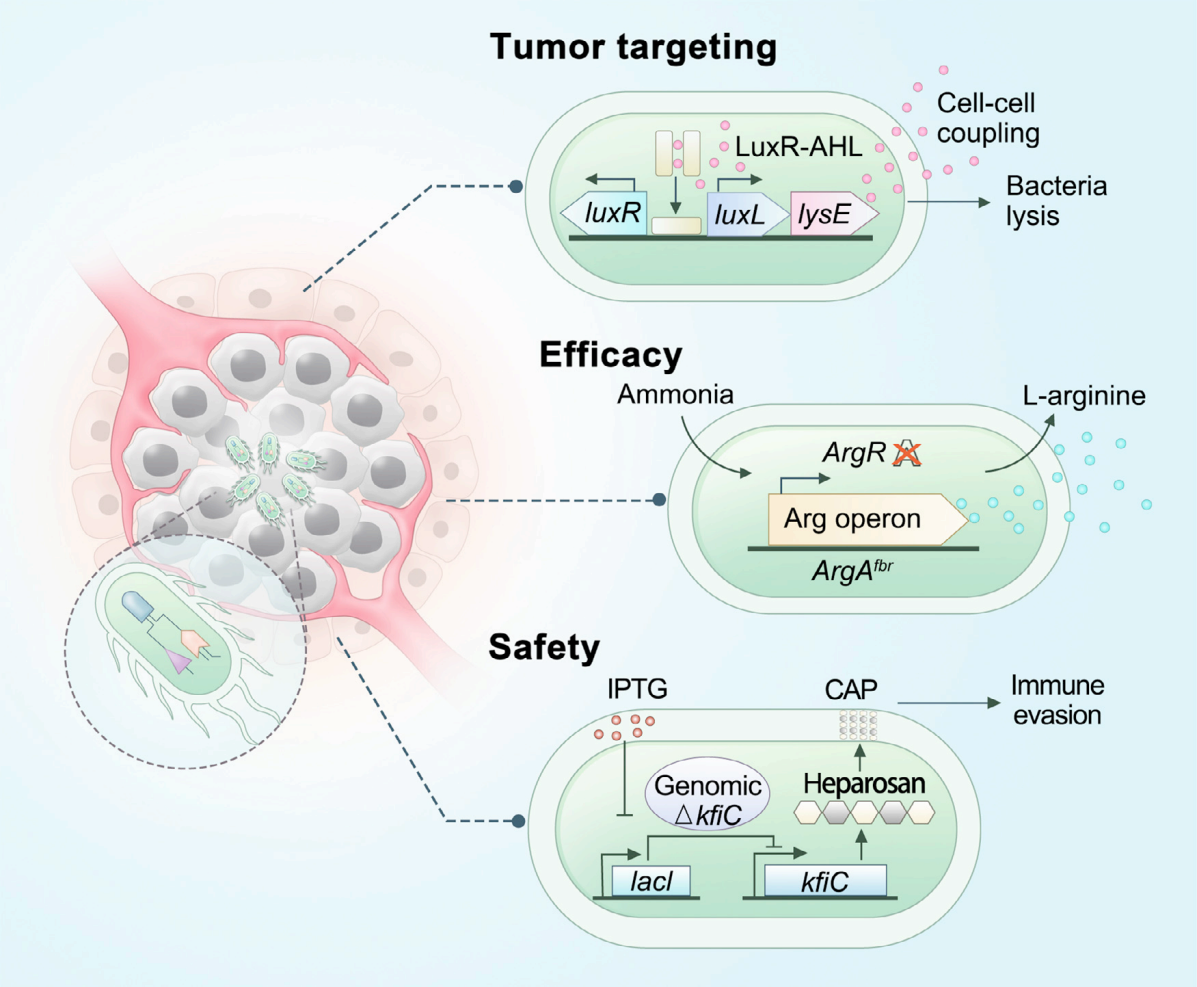
**Artificial antitumor bacteria.** Synthetic biology techniques are being employed to enhance the spatial and temporal controllability of antitumor bacteria, which can largely improve their safety and efficacy. A quorum-sensing gene circuit was designed to generate synchronized cell lysis and antitumor drug delivery, which can thus maintain the intratumoral bacterial population at a defined size and minimize the potential risk [10]. An engineered *Escherichia coli* was established with the ability of locally and continuously produce l-arginine in tumor tissues [12]. A genetically encoded microbial encapsulation system with tunable and dynamic expression of surface capsular poly-saccharides was constructed to precisely control bacterial immunogenicity and survivability *in vivo*[13]. CAP, capsular polysaccharides; IPTG, isopropyl-β-_d_-thiogalactoside.

In addition to utilizing wild types, bacteria can also be programmed through genetic engineering to the second-generation antitumor bacteria (Fig. 2). Synthetic biology techniques are being employed to enhance the spatial and temporal controllability of antitumor bacteria, which can largely improve their safety and efficacy. Firstly, genetic engineering can further improve the bacterial tumor targeting capacity. A quorum-sensing gene circuit was designed to generate synchronized cell lysis and antitumor drug delivery, so that the intratumoral bacterial population can be maintained at a defined size and minimize the potential risk [10]. Secondly, some additional antitumor function modules can be assembled to bacteria by applying genetic engineering. For example, a probiotic bacteria system was designed to continuously *in situ* produce and release checkpoint blockage antibodies within tumors [11]. In addition, an engineered *Escherichia coli* was established with the ability of locally and continuously produce l-arginine in tumor tissues [12]. Intratumoral colonization of these bacteria could increase the tumor concentration of l-arginine and the infiltration of T cells, thereby exhibiting remarkable synergistic effect with PD-L1 blocking antibodies. Thirdly, safety modules also can be assembled to bacteria by genetic engineering to reduce its toxicity. A genetically encoded microbial encapsulation system with tunable and dynamic expression of surface capsular poly-saccharides was constructed to precisely control bacterial immunogenicity and survivability *in vivo* [13]. This programmable encapsulation system was able to enhance the therapeutic efficacy and safety of living engineered bacteria for treating cancer. With more rationally design in future, the artificial antitumor bacteria will hopefully become a powerful weapon in the fight against cancer.

## 4 Current challenges and future developments

Once perfected, intratumoral microorganisms are promised to become essential clinical tools enabling multiple functions that are unachievable by other therapies. Therefore, it is meaningful to explore the detection, identification, and functional analysis of intratumoral microorganisms. However, the existing technologies have limited the scope and depth of studies on intratumoral microorganisms. First of all, characterization of intratumoral microorganisms still remains challenging due to their low biomass levels. Thus, the detection methods with low thresholds need to be developed to improve the sensitivity. In addition, environmental contamination greatly hampers the detection of intratumoral microorganisms. Therefore, besides improving experimental design, it is also important to develop perfect decontamination algorithms for data analysis. Finally, current studies mostly focus on the correlation between intratumoral microorganisms and tumor biology, and the high difficulty of *in vitro* cultivation of intratumoral microorganisms limited the mechanism study. Some new cultivation technologies can be employed for culturing intratumoral microorganisms, such as cultivation using microfluidic systems to mimic TME and culturomics which can use multiple conditions to culture different types of intratumoral microorganisms.

It has been found that regulating the gut microbiome can promote tumor therapy, thus studying and manipulating the intratumoral microbiome is also expected to enhance clinical responses to cancer therapy. In terms of the engineering of artificial antitumor bacteria, novel therapeutic targets need to be discovered. For example, bacteria can be programmed to secrete small molecular immunomodulators to improve immunotherapy, such as _S_-2-hydroxyglutarate [14]. In addition, most of the current artificial antitumor bacteria are based on model bacteria (*e.g., E. coli* Nissle 1917, attenuated *Salmonella, Lactobacilli, Bifidobacterium, and Akkermansia muciniphila*), and using the highly abundant intratumoral microorganisms and engineering them for fighting tumors maybe a better option. Moreover, in addition to studying the interactions between microorganisms and tumor cells (or immune cells), it is also very important to understand the interactions between different microorganisms inside one tumor. For example, metabolites excreted from one kind of bacteria can possibly influence the growth of other kinds of bacteria. Finally, despite the high diversity and availability of microorganisms within tumors, and substantial advances have been made in the progression of bacteria cancer therapies (*e.g.*, attenuated *Mycobacterium bovis* strain derived Bacillus Calmette-Guerin and *E. coli* Nissle strain derived SYN1891), biocontainment and safety concerns need to be evaluated during clinical translation. If employing microorganisms for fighting cancers, many problems still limit their development and applications, such as the unclear antitumor mechanism, toxicity, and weak spatiotemporal controllability. Engineering *in situ* intratumoral microorganisms through synthetic biology techniques, or combining artificial antitumor bacteria with immunotherapy or other more-powerful antitumor agents, may unlock the next generation of precision cancer diagnosis and therapy.

## CRediT authorship contribution statement

**W. L.** analyzed the literatures, conceived the manuscript structure, and wrote the manuscript. **X. M.** edited the figures and participated in the writing and revision of the manuscript. **X. Z.** inspired the main ideas of this perspective article, supplied the relevant literatures, and revised the manuscript in detail.

## Declaration of competing interest

The authors declare that they have no conflicts of interest in this work.
